# Coral Holobionts Possess Distinct Lipid Profiles That May Be Shaped by Symbiodiniaceae Taxonomy

**DOI:** 10.3390/md20080485

**Published:** 2022-07-28

**Authors:** Tatyana V. Sikorskaya, Ekaterina V. Ermolenko, Kseniya V. Efimova, Ly T. P. Dang

**Affiliations:** 1 A.V. Zhirmunsky National Scientific Center of Marine Biology, Far Eastern Branch, Russian Academy of Sciences, ul. Palchevskogo 17, 690041 Vladivostok, Russia; ecrire_711@mail.ru (E.V.E.); xengen88@gmail.com (K.V.E.); 2 Institute of Natural Products Chemistry, Vietnam Academy of Science and Technology, 18 Hoang Quoc Viet, Hanoi 100000, Vietnam; phuongly1412@gmail.com; 3 Graduate University of Science and Technology, Vietnam Academy of Science and Technology, Hanoi 100000, Vietnam

**Keywords:** corals, thylakoid, Symbiodiniaceae, lipidome, mass-spectrometry, genetic analysis

## Abstract

Symbiotic relationships are very important for corals. Abiotic stressors cause the acclimatization of cell membranes in symbionts, which possess different membrane acclimatization strategies. Membrane stability is determined by a unique lipid composition and, thus, the profile of thylakoid lipids can depend on coral symbiont species. We have analyzed and compared thylakoid lipidomes (mono- and digalactosyldiacylglycerols (MGDG and DGDG), sulfoquinovosyldiacylglycerols (SQDG), and phosphatidylglycerols (PG)) of crude extracts from symbiotic reef-building coral *Acropora* sp., the hydrocoral *Millepora platyphylla*, and the octocoral *Sinularia flexibilis*. *S. flexibilis* crude extracts were characterized by a very high SQDG/PG ratio, a DGDG/MGDG ratio < 1, a lower degree of galactolipid unsaturation, a higher content of SQDG with polyunsaturated fatty acids, and a thinner thylakoid membrane which may be explained by the presence of thermosensitive dinoflagellates *Cladocopium* C3. In contrast, crude extracts of *M. platyphylla* and *Acropora* sp. exhibited the lipidome features of thermotolerant Symbiodiniaceae. *M. platyphylla* and *Acropora* sp. colonies contained *Cladocopium* C3u and *Cladocopium* C71/C71a symbionts, respectively, and their lipidome profiles showed features that indicate thermotolerance. We suggest that an association with symbionts that exhibit the thermotolerant thylakoid lipidome features, combined with a high Symbiodiniaceae diversity, may facilitate further acclimatization/adaptation of *M. platyphylla* and *Acropora* sp. holobionts in the South China Sea.

## 1. Introduction

Coral, as a holobiont, forms complex interactions with a wide range of microorganisms that constitute an important part of the entire metaorganism [1]. The best-known coral symbionts are intracellular microalgae (dinoflagellates of the family Symbiodiniaceae) [1]. In response to an increase in sea surface temperature and solar radiation, coral symbionts can be completely or partially expelled, which results in coral bleaching. This process is a major cause of coral reef destruction [2] and, therefore, a subject of intensive research worldwide. Indo-Pacific coral reefs are spread over a vast ocean area. The South China Sea (SCS) is a region in the central Indo-Pacific [3] that, despite being adjacent to the western border of the Coral Triangle, a recognized center of maximum marine biodiversity, has received much less scientific and conservation attention [4]. Nevertheless, SCS coral reefs also exhibit an unexpectedly high biodiversity [5].

Reef-building scleractinian corals (Anthozoa), producing calcium carbonate (CaCO_3_) skeletons, are a key habitat-forming group in tropical reefs that provides shelter for many other marine organisms [6]. Approximately 757 species of reef-building corals are currently reported for shallow-water reefs of the Indo-Pacific [7]. Symbiotic taxa from the family Milleporidae (Hydrozoa) are also important members of reef-building cnidarians. The genus *Millepora* comprises around 16 species distributed over tropical/subtropical seas worldwide [8]. Octocorals are considered the second most common group of macrobenthic animals after the reef-building stony corals in many shallow-water Indo-Pacific reefs [9]. Octocorallia (Anthozoa) can be recognized by their distinctive morphological features: polyps with eight-fold symmetry and an internal skeleton composed of microscopic calcareous sclerites [10]. Alcyonacea is the most speciose order of Octocorallia, encompassing the vast majority of octocoral taxa in tropical coral reefs [9].

On the basis of nuclear ribosomal DNA (rDNA) (the ribosomal large subunit (LSU) 28S rDNA and small subunit (SSU) 18S rDNA, and the internal transcribed spacer (ITS) regions) and plastid 23S rDNA marker regions, the family Symbiodiniaceae was divided into nine phylogenetic clades (A to I) and then further subdivided into 15 genus-level lineages [11,12,13,14,15,16]. Symbiotic corals are most commonly associated with Symbiodiniaceae of the genera *Symbiodinium* (clade A), *Breviolum* (clade B), *Cladocopium* (clade C), *Durusdinium* (clade D), *Effrenium* (clade E), *Fugacium* (clade F), and *Gerakladium* (clade G) [16]. Various Symbiodiniaceae genera differ in their degree of tolerance to increased light and temperature conditions [2,17,18,19]. For example, *Symbiodinium thermophilum* is a locally abundant Symbiodiniaceae species in reef-building corals of the southern Persian Gulf (Arabian Sea), a habitat distinguished by a high water temperature [20]. The thermotolerant *Durusdinium trenchii* is widely distributed in ocean waters with abnormally high temperatures [21], while common symbionts of the genus *Cladocopium* are known to be more thermosensitive [21]. Nevertheless, a culture of *Cladocopium* C1 or *Breviolum psygmophilum* (1046) in laboratory experiments exhibited better photophysiology compared to other Symbiodiniaceae species [22,23]. These adaptations translate into different properties for the coral host [24,25]. 

Another photosynthesizing coral symbiont is an endolithic alga of the genus *Ostreobium* (Bryopsidales, Ulvophyceae) [26]. It absorbs light of a wavelength not used by symbiotic dinoflagellates. *Ostreobium* carries out oxygenic photosynthesis even under extreme low light conditions [27]. It has been assumed to replace the function of symbiotic dinoflagellate during bleaching events [28]. An environmental genome survey has shown a possible co-evolution between *Ostreobium* and both coral and dinoflagellates [26]. In contrast to that of Symbiodiniaceae, the relationship between the *Ostreobium* and a cnidarian host are far from being completely understood. 

Lipids play an essential role in metabolism and maintaining health of corals [29,30]. The lipid composition of each taxon largely depends on its genetic capability of synthesizing certain fatty acids (FA). Therefore, FAs are successfully used in chemical systematics of various taxonomic groups including bacteria, fungi, macro- and microalgae, and corals [31,32,33]. Several classes of glycolipids such as sulfoquinovosyldiacylglycerol (SQDG), monogalactosyldiacylglycerol and digalactosyldiacylglycerol (MGDG and DGDG), and phosphorus-containing class phosphatidylglycerol (PG) are essential components of biomembranes of the photosynthetic apparatus in plants and form a basis of symbiotic dinoflagellate lipids [34,35,36]. Their relative proportions are stable, indicating the presence of mechanisms responsible for and controlling a steady state (homeostasis) of the membrane lipidome in thylakoids, in certain environmental and physiological contexts [37]. These lipid classes, as well as some polyunsaturated fatty acids (PUFA), which comprise acyl groups of MGDG and DGDG, are considered the major lipid biomarkers of coral endosymbionts [38,39,40]. There are few studies on the molecular species profile of certain thylakoid lipid classes in coral endosymbionts [37,38]. Studies of thylakoid membranes are mainly based on the FA composition of total thylakoid lipids (SQDG, MGDG, and DGDG) [38,39,40]. SQDG mainly contains saturated acyl chains [37], while galactolipids are highly unsaturated [37], and their combined analysis may be challenging. 

Since it was previously shown that the composition of marker lipid classes (PG, MGDG, DGDG, and SQDG) of the Symbiodiniaceae genera with different thermal sensitivities has distinctive features [41,42], the profile of thylakoid lipids can depend on species of photosynthetic coral symbionts. In order to test this hypothesis, we collected colonies of the major taxonomic groups of SCS corals: the reef-building coral *Acropora* sp., the hydrocoral *Millepora platyphylla*, and the octocoral *Sinullaria flexibilis*. We investigated the molecular genetic diversity of symbiotic dinoflagellates and *Ostreobium* communities associated with colonies of this coral species on the basis of the nuclear ITS1-5.8S-ITS2 rRNA region, ITS2 region, 28S rRNA gene, and plastid 23S rRNA (pDNA) sequences. Using chromatography with high resolution mass-spectrometry, we identified lipidome profiles of the thylakoid membrane of symbiotic coral dinoflagellates (PG, MGDG, DGDG, and SQDG). Then, we compared the obtained genetic and lipidome data. 

## 2. Results

### 2.1. Genetic Identification of Coral Endosymbionts

To identify the intragenomic variants for the internal transcribed spacer-2 (ITS2) region, we determined the SymPortal [43] profiles for the Symbiodiniaceae community in corals. Our study shows the presence of multiple Symbiodiniaceae types in all of the *Acropora* sp. and *M. platyphylla* colonies and in three colonies of *S. flexibilis* (Table 1). 

The most common types among *Acropora* sp. colonies were *Breviolum* B1 (*B. minutum*) and *Cladocopium* C3u, occurring together in the same colonies. *Symbiodinium* A1a type was found in two colonies of *Acropora*, with *Breviolum* and *Cladocopium* occurring in the same colonies. Some of Symbiodinaceae taxa were found in a single colony, for example, C66 and C3sg in A2 colony. The rare C50bn type was found in two (A4 and A5) of five *Acropora* sp. colonies. We also found a sequence of green algae, *Ostreobium* sp. (Bryopsidales, Ulvophyceae), in the A4 colony. The 23S rRNA gene sequence shared a 99% similarity to the published sequence of *Ostreobium* sp. HV05042 [44] with only a 1-bp difference. 

The *Cladocopium* C71/C71a type was common across *M. platyphylla* colonies. *Durusdinium* D1 type (*D. trenchii*) was found in four colonies of *M. platyphylla*, with *Cladocopium* in the same colony. Type C3 was found only in the M5 colony mixed with C3sg, C71/C71a, and D1. We also found sequences of green algae *Ostreobium* sp. (Bryopsidales, Ulvophyceae) in the M2–M5 colonies. The 23S rRNA gene sequences in the M2–M5 colonies shared a 100% identity with the same sequence from the *Acropora* colony A4 and a 99% similarity to the published sequence of *Ostreobium* sp. HV05042.

Most Symbiodiniaceae species found in the *S. flexibilis* colonies were of two main types, C3 and C71/C71a. The *Breviolum* B1 type (*B. minutum*) was found only in the S1 colony, and the C3sg type was in the S4 coral colony. Some of the colonies were associated with one Symbiodiniaceae type. S2 and S3 harbored the C3 type, whereas colonies of S1, S4, and S5 were associated with multiple types: B1 + C3 + C71/C71a, C3 + C3sg + C71/C71a, and C3 + C71/C71a, respectively. 

### 2.2. Thylakoid Membrane Lipidome of Coral Endosymbionts

#### 2.2.1. Phospholipid PG and Glycolipid SQDG

**Figure 1:**
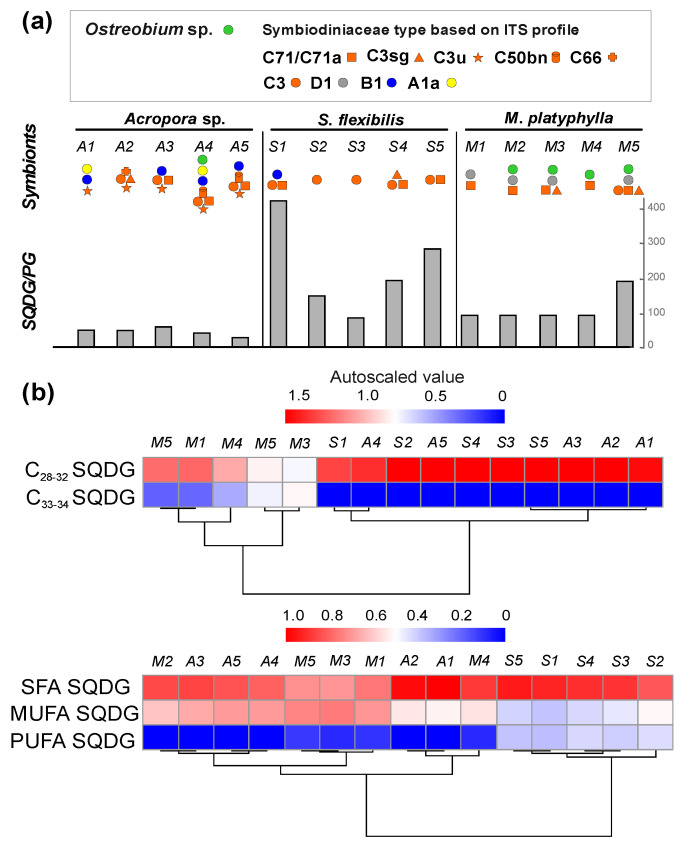
The lipidome features of the coral colonies *Acropora* sp. (A1–A5), *Sinularia flexibilis* (S1–S5), and *Millepora platyphylla* (M1–M5). (**a**) A ratio of total sulfoquinovosyldiacylglycerol (SQDG) to total phosphatidylglycerol (PG). (**b**) A heat map of SQDG molecular species grouped on the basis of chain length (C_28–32_ and C_33–34_) and fatty acids with different unsaturation degree (saturated fatty acids (SFA), monounsaturated fatty acids (MUFA), and polyunsaturated fatty acids (PUFA)) with a clustering (tree clustering, wards method, and Euclidean distances). The scale bar above the heatmap(s) represents the arcsine-transformed relative abundance of lipid content in the samples. Symbiodiniaceae community in corals was determined based on the internal transcribed spacer-2 (ITS2) region. Symbiodiniaceae type: “A1a”—*Symbiodinium*; “B1”—*Breviolum*; “C3”, “C3sg”, “C3u”, “C50bn”, “C66”, “C71/C71a”—*Cladocopium*; “D1”—*Durusdinium*. The green algae *Ostreobium* sp. (Bryopsidales, Ulvophyceae) in corals was determined based on the 23S rRNA gene sequences.

Coral lipid extracts contained small amounts of PG (0.003–0.147% of lipid extracts), especially in *S. flexibilis*, where only a single molecular species (PG 16:2/20:2) was detected (Table S1). The *Acropora* sp., *S. flexibilis*, and *M. platyphylla* extracts contained SQDG at 0.50–13.86% of lipid extracts (Table S1). The SQDG/PG ratio varied between corals. The highest SQDG/PG ratio was observed in *S. flexibilis* (from 415.95 to 82.32). In *Acropora* sp. and *M. platyphylla*, the SQDG/PG ratios were 25.71–46.85 and 40.92–186.68, respectively (Figure 1a, Table S1). 

In this study, 14 molecular species of SQDG were identified (Figure 1b, Table S1). They were grouped on the basis of chain length and unsaturation degree of FA residues: C_28–32_ SQDG (with C_14,16_ FAs), C_33–34_ SQDG (with C_17,18_ FAs), SFA SQDG (with saturated FAs), MUFA SQDG (with monounsaturated FAs), and PUFA SQDG. The total C_28–32_ SQDG content was higher in the *Acropora* sp. and *S. flexibilis* lipid extracts than in the *M. platyphylla* extracts. A higher content of C_33–34_ FA SQDG species (16:0/17:0, 16:0/18:3, 16:0/18:2, 16:0/18:1, and 16:0/18:0) was observed in the *M. platyphylla* extracts. These components were not detected in the *Acropora* sp. and *S. flexibilis* samples (Table S1). The content of MUFA SQDG in *S. flexibilis* lipids was significantly lower than that in *M. platyphylla* and *Acropora* sp. lipids (HSD test, *p* < 0.1). Moreover, a high content of PUFA SQDG was observed in *S. flexibilis* (16.35 ± 1.85% of total SQDG). These molecular species were absent in *Acropora* sp. and were present in *M. platyphylla* only in small amounts (0.82 ± 0.51% of total SQDG) (Table S1). A cluster analysis of SQDG with different chain lengths split the coral species into two clusters: (1) *M. platyphylla* and (2) *S. flexibilis* and *Acropora* sp. colonies. A cluster analysis of SQDG with different unsaturation degrees split the coral species into two clusters: (1) *S. flexibilis* and (2) *Acropora* sp. and *M. platyphylla* colonies (Figure 1b). 

#### 2.2.2. Galactolipids MGDG and DGDG

**Figure 2:**
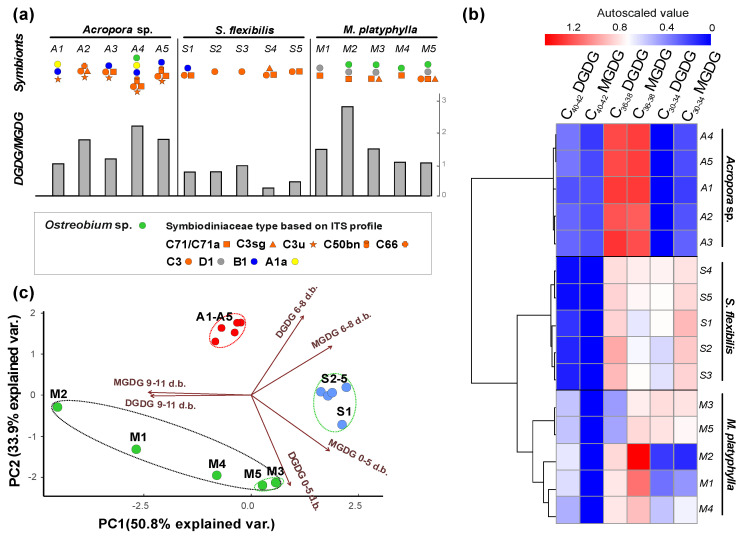
The lipidome features of the coral colonies *Acropora* sp. (A1–A5), *Sinularia flexibilis* (S1–S5), and *Millepora platyphylla* (M1–M5). (**a**) A ratio of total digalactosyldiacylglycerol (DGDG) to total monogalactosyldiacylglycerol (MGDG). (**b**) A heat map of galactolipid molecular species grouped on the basis of chain length (C_30–34_, C_36–38_, and C_40–42_) with a clustering (tree clustering, wards method, and Euclidean distances). The scale bar above the heatmap(s) represents the arcsine-transformed relative abundance of lipid content in the samples. (**c**) A principal component analysis of the composition of galactolipid molecular species with different unsaturation degrees (with 0–5 double bonds (MGDG 0–5 d.b. and DGDG 0–5 d.b.), 6–8 double bonds (MGDG 6–8 d.b. and DGDG 6–8 d.b.), and 9–11 double bonds (MGDG 9–11 d.b. and DGDG 9–11 d.b.)). The dotted line outlines clusters (tree clustering, wards method, and Euclidean distances). Symbiodiniaceae community in corals was determined based on the internal transcribed spacer-2 (ITS2) region. Symbiodiniaceae type: A1a”—*Symbiodinium*; “B1”—*Breviolum*; “C3”, “C3sg”, “C3u”, “C50bn”, “C66”, “C71/C71a”—*Cladocopium*; “D1”—*Durusdinium*. The green algae *Ostreobium* sp. (Bryopsidales, Ulvophyceae) in corals was determined based on the 23S rRNA gene sequences.

The *Acropora*, *S. flexibilis*, and *M. platyphylla* extracts contained MGDG at 1.23–5.32% and DGDG at 0.86–6.48% of lipid extracts, respectively. The DGDG/MGDG ratio varied between corals. The lowest DGDG/MGDG ratio was observed in *S. flexibilis* (0.25–0.95). In *Acropora* sp. and *M. platyphylla*, the DGDG/MGDG ratios were 1.01–2.19 and 1.04–2.95, respectively (Figure 2a, Table S1).

A total of 26 molecular species of MGDG and 24 molecular species of DGDG were identified (Table S1). These were grouped on the basis of chain length of FA residues: C_30–34_ MGDG and DGDG (with C_16,18_ FAs), C_36–38_ MGDG and DGDG (with C_18,20_ FAs), and C_40–42_ MGDG and DGDG (with C_18,20,22_ FAs) (Figure 2b, Table 2). C_36–38_ MGDG and DGDG were most abundant in the *Acropora* sp. colonies (89.08 ± 2.87 and 89.77 ± 1.98% of total MGDG and DGDG, respectively). In the *S. flexibilis* and *M. platyphylla* colonies, C_30–34_ and C_36–38_ MGDG and DGDG were most abundant in comparable proportions: in *S. flexibilis*, MGDG accounted for 56.18 ± 4.60 and 43.61 ± 4.37%, and DGDG accounted for 40.51 ± 6.02 and 58.71 ± 5.83%, respectively. In the *M. platyphylla* colonies M3-M5, MGDG accounted for 45.73 ± 5.71 and 54.27 ± 6.17%, DGDG accounted for 43.78 ± 14.10 and 29.29 ± 17.44% of total DGDG, respectively. C_40–42_ MGDG were detected only in the *Acropora* sp. colonies (4.43 ± 0.96% of total MGDG). C_40–42_ DGDG were detected in all of the coral colonies studied. Their content was significantly higher in *M. platyphylla* colonies (29.86 ± 5.62% of total DGDG) relative to the *S. flexibilis* and *Acropora* sp. colonies (0.98 ± 0.94 and 8.83 ± 2.29% of total DGDG, respectively) (Table S1). A cluster analysis of MGDG and DGDG with different FA lengths split the coral colonies into two clusters: (1) *Acropora* sp. colonies and (2) *S. flexibilis* and *M. platyphylla* colonies (Figure 2b).

The MGDG and DGDG molecular species were grouped on the basis of the unsaturation degree of molecules: MGDG and DGDG with 0–5 double bonds, MGDG and DGDG with 6–8 double bonds, and MGDG and DGDG with 9–11 double bonds (Figure 2c; Table S1). A PCA analysis of MGDG and DGDG molecular species with different unsaturation degrees (with PC1 constituting 58% of variations; PC2, 33%; and eigenvalue > 1) showed a shift of *S. flexibilis* colonies along the PC1 axis (PC1 axis correlated with MGDG and DGDG with 9–11 double bonds (*r* = −0.53 and *r* = −0.51, respectively) and MGDG with 0–5 and 6–8 double bonds (*r* = 0.40 and *r* = 0.42, respectively)) (Figure 2c and Figure S1; Table S2). This shift was driven by a higher content of MGDG with 0–5 and 6–8 double bonds (34.83 ± 5.31 and 47.68 ± 5.06% of total MGDG, respectively) than that in *M. platyphylla* colonies, and lower contents of MGDG and DGDG with 9–11 double bonds than these in *M. platyphylla* and *Acropora* sp. colonies. The shift of the *Acropora* sp. colonies along the PC2 axis (PC2 axis correlated with DGDG with 0–5 and 6–8 double bonds (*r* = −0.64 and *r* = 0.56, respectively) and MGDG with 0–5 and 6–8 double bonds (*r* = −0.39 and *r* = 0.34, respectively)) was driven by a higher content of MGDG and DGDG with 6–8 double bonds (49.01 ± 2.17 and 45.15 ± 6.89% of total MGDG and DGDG, respectively), a lower content of MGDG with 0–5 double bonds (5.14 ± 1.48% of total MGDG), and the lack of DGDG with 0–5 double bonds. On the basis of these parameters, the *Acropora*, *M. platyphylla*, and *S. flexibilis* colonies formed separate clusters (Figure 2c and Figure S2). The *M. platyphylla* colonies were not grouped into common clusters with *Acropora* or *S. flexibilis*. The contents of MGDG and DGDG with different unsaturation degrees were not similar between the *M. platyphylla* colonies (Table 2). M3 and M5 were similar in contents of MGDG and DGDG with different unsaturation degrees. MGDG with 6–8 double bonds (41.28 ± 1.65% of total MGDG) and DGDG with 0–5 double bonds (57.82 ± 1.30% of total DGDG) were most abundant. M1, M2, and M4 contained a higher percentage of MGDG (90.18, 82.82, and 43.90% of total MGDG, respectively) and DGDG (90.18, 82.82, and 63.21% of total DGDG, respectively) with 9–11 double bonds. The lowest percentage of MGDG and DGDG with 0–5 double bonds (0.32 and 2.61% of total MGDG and DGDG, respectively) was recorded from M1.

## 3. Discussion

Symbiotic relationships are of crucial importance for a coral holobiont and its adaptation to changing environmental conditions. Various corals have different degrees of specificity to certain types of Symbiodiniaceae. The species-specific interactions between algal endosymbionts and coral hosts are determined by the glycan–lectin interactions [45]. Thus, corals contain one main Symbiodiniaceae genus and several additional as a way of adaptation [46]. According to a genetic analysis, *Acropora* sp. (A1–A5), *M. platyphylla* (M1–M5), and *S. flexibilis* (S1, S4, S5) contained several species of symbionts (symbiotic dinoflagellates and *Ostreobium* sp.). A cluster analysis of thylakoid lipids showed that colonies were clustered depending on the coral species. This probably indicates that each coral species hosted one main Symbiodiniaceae species that contributed more to the lipidome profile of thylakoid membrane. All of the studied *Acropora* sp. colonies hosted *Cladocopium* C3u. As was shown earlier, in the corals *A. formosa*, *A. corymbosa*, and *A. digitifera*, the *Cladocopium* C3u is one of the main symbionts [47,48,49]. The *S. flexibilis* colonies contained *Cladocopium* C3, which is a common species for corals and was previously found in corals of this genus [42,50]. Only *Cladocopium* C71/C71a was found in all the examined colonies of *M. platyphylla*. 

### 3.1. Thylakoid Lipidome Features of Coral Endosymbionts

The cell membrane acclimatization in biological systems occurs under abiotic stressors such as elevated temperature and high solar irradiance [51,52]. The membrane stability is determined by a unique lipid composition, or lipidome. The lipidome of thylakoid membranes of each photosynthetic symbiont species is characterized by its distinguishing features [41,42]. Lipidome remodeling is a way of acclimatization of the photosynthesis apparatus exposed to adverse factors. Different species may have their own strategies for membrane acclimatization, with their efficiency depending on the initial lipidome. In the present work, it was shown that *Acropora*, *M. platyphylla*, and *S. flexibilis* colonies formed distinct clusters based on various parameters such as degree of unsaturation and chain length of SQDG and galactolipids. These parameters can determine the membrane properties [53]. The lipid unsaturation degree influences membrane viscosity during the acclimatization to stressful temperatures, while the lipid head groups can impart curvature or surface charge [54].

#### 3.1.1. Lipid Head Groups

In an organism under stable environmental conditions, the membrane lipidome in thylakoids is in a steady state (homeostasis), which is manifested as a constant ratio of thylakoid lipids. Anionic phosphoglycerolipid PG is considered a vital lipid, mainly for its role as a cofactor of photosystems. The anionic glycerolipid SQDG with a sulfur-containing polar head interacts with photosynthetic proteins and some annexins [55]. Under normal conditions, SQDG is dispensable [56] and SQDG appears as a sort of stand-in actor performing the PG functions when phosphate is limited [37]. The SQDG/PG ratio has a constant value for a particular photosynthetic organism and can be considered as its characteristic feature. It is probable that such a feature as the very high SQDG/PG ratio that distinguishes the octocoral *S. flexibilis* may be caused by the presence of the thermosensitive *Cladocopium* C3. Nonetheless, *Cladocopium* C3 was also present in *Acropora* (A2, A3, A4 and A5) and *M. platyphylla* (M5) colonies that were characterized by a low SQDG/PG ratio. Since our work only established the presence or absence, but not the relative abundance, of certain algae symbionts, we can only hypothesize, but not proof, that *Cladocopium* C3 was the main symbiont species in *S. flexibilis*, while it was an additional, possibly less abundant symbiont type in *Acropora* and *M. platyphylla*. 

Galactoglycerolipids are the most abundant lipids in photosynthetic membranes. Two neutral major classes, MGDG and DGDG, contain one or two galactose residues in their polar head, respectively [37]. These lipids have contrasting biophysical properties, with DGDG being bilayer-forming and MGDG non-bilayer lipids [57]. The DGDG/MGDG ratio is crucial for proper physiological functioning of thylakoid membrane [58]; it remains stable as plants grow under favorable controlled conditions [37]. Recently, it has been shown that the DGDG/MGDG ratio recorded from thermosensitive *Cladocopium* C3 is significantly lower compared to that of thermotolerant *D. trenchii* (both hosted by *A. valida*) [41]. In the thermotolerant symbiotic dinoflagellate *Symbiodinium microadriaticum*, the DGDG/MGDG ratio increases under heat stress [35]. In our work, the DGDG/MGDG ratio in *S. flexibilis* was lower, in contrast to *Acropora* sp. and *M. platyphylla*, where this ratio was higher. Thus, such a feature of *S. flexibilis* as DGDG/MGDG < 1 is likely to be caused by the presence of the thermosensitive *Cladocopium* C3. Having a higher DGDG/MGDG ratio (>1), symbionts of *Acropora* sp. and *M. platyphylla* may exhibit features of thermotolerant symbiotic dinoflagellates.

#### 3.1.2. Lipid Acyl Chains: Degree of Unsaturation 

The most obvious basic role of polar glycerolipids in thylakoids is to constitute a lipophilic matrix in which photosystems are embedded. The lipid matrix should allow the lateral diffusion of the photosystems. Due to the overcrowding of protein complexes in photosynthetic membranes, the lateral mobility might be relatively limited. The presence of multiple double bonds in FAs of galactoglycerolipids may ease the lateral dynamics in this packed environment [37]. 

The separation of *S. flexibilis* colonies as a single cluster associated with the lowest degree of MGDG and DGDG unsaturation may be caused by the presence of *Cladocopium* C3 type. Earlier, this galactolipid feature was demonstrated for *Cladocopium* C3 host-associated by *A. valida* and *S. heterospiculata* [41,42]. A high degree of unsaturation of MGDG and DGDG is reported to be more typical of the thermotolerant *D. trenchii* hosted by *A. valida* and *Palythoa tuberculosa* [41,42]. A genetic analysis did not show the presence of *D. trenchii* in the *Acropora* symbiotic community. Apparently, this galactolipid feature may be determined by the presence of such symbionts as *Cladocopium* C3u that were found in all five *Acropora* colonies. A large variation in the degree of unsaturation of MGDG and DGDG was observed among the colonies of *M. platyphylla*. The presence of *Cladocopium* C3sg in the M3 and M5 colonies of *M. platyphylla* may result in a lower galactolipid unsaturation in contrast with M1, M2, and M4 colonies with an increased degree of galactolipid unsaturation. Probably, in addition to *Cladocopium* C71/C71a, this species of symbionts can significantly contribute to the thylakoid lipidome of the *Millepora* colonies M3 and M5. On the other hand, such symbiont species as *Cladocopium* C3, C71/C71a and C3sg were found simultaneously in *Acropora*, *S. flexibilis* and *M. platyphylla*. Therefore, the pattern of glycolipid composition differences between corals may be more complex.

SQDG of free-living and hosted dinoflagellates were previously reported to be saturated or monounsaturated [34,39,42,59]. Rosset et al. [41] suggested the central role of the SQDG saturation in response to the temperature stress, as the unsaturation degree was already reduced in *D. trenchii* at ambient temperature, and the difference from *Cladocopium* C3 further increased under temperature stress. In our work, the presence of *Cladocopium* C3 in the octocoral *S. flexibilis* correlated with an increased content of SQDG with PUFAs. Previously, the same feature was shown for *Cladocopium* C3 hosted by *A. valida* [41]. In contrast, SQDG with PUFAs were either absent or present in trace amounts in *Acropora* sp. and *M. platyphylla*. This is a characteristic feature of the thermotolerant *D. trenchii* [41,42].

#### 3.1.3. Lipid Acyl Chains: Chain Length 

The composition of bilayer-forming DGDG in the corals under study varied. Both short- and medium-length DGDG were present in the *S. flexibilis* colonies. In contrast to the *Acropora* sp. colonies that contained medium-chain DGDG, the *M. platyphylla* colonies, in addition to short- and medium-chain DGDG, also contained long-chain DGDG. The length of the acyl group of membrane lipid molecules strongly influences the thickness of lipid bilayer [60]. Thus, the thylakoid membrane of *S. flexibilis* symbionts is probably thinner, in contrast to that of symbionts of *Acropora* sp. and *M. platyphylla*, whose lipidome features are similar to those of the thermotolerant *D. trenchii*. Numerous studies support a growing consensus that lipid bilayers can play an essential role in determining the function of membrane proteins [61], whose behavior can be severely compromised depending on the thickness and curvature of the lipid bilayer [62,63]. The bilayer thickness has been associated with other membrane functions such as pore formation [64] and passive permeation [65]. Since the DGDG molecule is known to be formed through the addition of a galactose unit to the MGDG molecule [36], patterns of the DGDG and MGDG compositions may be similar, as we observed in *S. flexibilis* and *Acropora* sp. On the contrary, MGDG and DGDG compositions were different in *M. platyphylla*. Long-chain MGDG were absent and the content of long-chain DGDG was above 25%. Probably, the MGDG chain length can play a different function in *M. platyphylla*. With forming reverse micelles, MGDG may be beneficial in highly curved membrane domains or in the vicinity of some large protein complexes [58].

The chain length of SQDG also varies between corals containing different symbionts. SQDG with longer chains were a characteristic feature of the symbionts hosted by *M. platyphylla*. The symbionts of *S. flexibilis* and *Acropora* sp. typically had SQDG with shorter chains. The chain lengths of MGDG, DGDG, and SQDG varied even within the same coral species. Differences between SQDG and other GL can probably be explained by the different locations of these GL in the thylakoid membrane. As has recently been shown for diatoms, the thylakoid membrane has a characteristic arrangement where six membranes compose a band of three thylakoids that span all along the plastid length [66]. The segregation is assumed to be based on differences in the lipid composition between the outer and inner membranes, with the outer ones specifically enriched in SQDG, and the inner membranes containing more MGDG [67].

### 3.2. Symbionts with Thermotolerant Features of Lipidome May Help Corals Adapt to Climate Change in the SCS

The Symbiodiniaceae exhibit a high genetic diversity with different physiological properties of thylakoid membrane within and between species, resulting in the acclimation of their photosynthetic performance to various temperature and light conditions. The functional differences between symbiotic dinoflagellates are also observed at lower taxonomic levels, e.g., among species [68]. To survive adverse environmental conditions, the flexibility of a coral host to associate with different Symbiodiniaceae endosymbionts can be critical [18,24,47]. For example, a wide biodiversity of symbionts has been previously shown for corals of the genus *Acropora* [48,69]. *Symbiodinium*, *Breviolum* [70,71,72,73,74], *Cladocopium* also with *Durusdinium* and *Geracladium* were found in corals of the *Acropora* genus [71,72,73,75]. In contrast to *S. flexibilis* and *M. platyphylla*, a high diversity of Symbiodiniaceae and complex “mosaic” patterns of associations with different symbiont types were observed across the studied *Acropora* colonies. Recently, it has been shown that this resistance/adaption strategy is characteristic of branched corals (*Acropora* and *Pocillopora*) that exhibit a high intra- and inter-species flexibility in their Symbiodiniaceae assemblages, while massive corals (*Porites*) exhibit a low flexibility and are environmentally highly resistant corals [76]. The enhanced symbiotic diversity observed in the *Acropora* colonies, represented by multiple genera, *Symbiodinium*, *Breviolum*, and *Cladocopium* (C3u, C71/C71a, C50bn, C3, and C3sg), is most likely an advantage in survival which leads to the acquisition of various tolerances and plasticity to environmental changes. The high diversity of Symbiodiniaceae, combined with the association with symbionts which exhibit thylakoid lipidome properties similar to those of the thermotolerant *D. trenchii*, can provide an opportunity for further acclimatization/adaptation of the *Acropora* sp. in the SCS.

Previous studies demonstrated that the Cnidaria–Symbiodiniaceae association is not stochastic, but mostly determined by host phylogeny and geography [77,78]. The coral genera that were found to maintain only a single Symbiodiniaceae species begin hosting more species with increase in latitude [48,79]. In our work, the coral colonies were collected in the SCS region at a lower latitude. Accordingly, these waters should be inhabited by corals with a lower symbiotic diversity. In our work, the colonies of the octocoral *S. flexibilis* and the hydrocoral *M. platyphylla* also showed a low biodiversity. Five colonies of *M. platyphylla* contained symbionts with thermotolerant lipidome features and, moreover, the colonies M1, M2, M3 and M5 additionally contained thermotolerant *Durusdinium*. Type D1, found in the *M. platyphylla* colonies, had not been recorded from *Millepora* before. Mutation rates in Symbiodiniaceae can be high, resulting in new variants of individual genotypes [80,81]. Natural selection leads to higher temperature tolerance inherited in symbiont populations [23]. By enhancing this selection in its symbiont population, the coral host can survive periods of warming [82]. Thus, the content of symbionts with thermotolerant features, which are most likely *Cladocopium* C71/C71a, as well as the ability to establish symbiosis with the thermotolerant *Durusdinium* can facilitate the survival of the hydrocoral *M. platyphylla* in the SCS. In contrast to the hydrocoral colonies, five colonies of *S. flexibilis* contained thermosensitive symbionts *Cladocopium* C3, while the colonies S1, S4, and S5 additionally contained C71/C71a, C3sg, and *Breviolum*. In the future, this specificity of *S. flexibilis* to the thermosensitive *Cladocopium* C3 type and the low symbiont diversity may pose a threat to the survival of this species in the SCS.

## 4. Conclusions

Genetic methods have become a standard, widely accepted approach to identifying organisms. However, lipid analysis is an additional independent method providing important results that are comparable with similar works based exclusively on analysis of FA profiles and occurrence of FAs in different lipid classes (lipidome). The use of a lipid profile as a chemotaxonomic biomarker alongside molecular genetics analyses can provide additional information on specific lipid compositions. Such polyphasic analysis can reveal new reliable chemotaxonomic markers for differentiating symbionts on a low taxonomic scale. Most likely, the thylakoid membrane lipidome is determined by the major symbiont species. Features of *S. flexibilis* such as a very high SQDG/PG ratio, a DGDG/MGDG ratio < 1, the lowest degree of galactolipid unsaturation, a higher content of SQDG with PUFAs, and a thinner thylakoid membrane may be explained by the presence of the thermosensitive dinoflagellate species *Cladocopium* C3. In contrast, symbionts of *M. platyphylla* and *Acropora* sp. exhibited thermotolerant lipidome features. Since each colony of *M. platyphylla* and *Acropora* sp. contained *Cladocopium* C3u and Cladocopium C71/C71a, respectively, these species could be responsible for the expression of thermotolerant lipidome features. Thus, there may be significant differences in thylakoid lipidome among the Symbiodiniaceae species within the same genus, likely associated with light affinity and temperature tolerance. In the future, a comprehensive comparative study of the thylakoid membrane structure and properties in symbionts at the intraspecific taxonomic level is necessary to fully understand the thylakoid lipidome features responsible for variations in thermotolerance.

## 5. Materials and Methods

### 5.1. Specimen Collection

Colonies of three tropical coral species, *Sinularia flexibilis* (class Anthozoa, subclass Octocorallia, order Alcyonacea, family Alcyoniidae), *Millepora platyphylla* (class Hydrozoa, subclass Hydroidolina, order Anthoathecata, family Milleporidae), and *Acropora* sp. (class Anthozoa, subclass Hexacorallia, order Scleractinia, family Acroporidae), were collected in the South China Sea. Specimens were sampled by SCUBA divers off three islands (Hon Mot: 12.181341° N, 109.276905° E; Hon Tre: 12.180763° N, 109.283778° E; and Hon Tam: 12.174820° N, 109.238961° E) at a depth of 3–8 m in April 2021 (Table S3). Environmental conditions at the sampling site are presented in Supplementary Materials. Colonies of the same coral species were sampled at a distance of at least three meters. The species were identified on the basis of arrangement of sclerites using a microscope. The colonies were carefully cleaned of non-coral debris. The specimens collected were placed immediately into tanks with seawater at the collection site and transported to the laboratory. Five different colonies of each coral species were used for lipid and molecular genetics analyses. 

### 5.2. Molecular Genetics Analysis

A total of 15 coral colonies representing three coral species (*n* = 5 per species) were collected. Extraction of DNA from ethanol-fixed tissue samples was carried out according to the guanidine thiocyanate-based protocol published previously [52]. Amplification reactions were performed on a thermal cycler (Applied Biosystems Veriti 96 Well, Waltham, MA, USA) using an iProof high-fidelity PCR master mix (Bio-Rad, München, Germany) according to the manufacturer’s protocol.

The presence of symbiont types was verified by PCR screening using *Symbiodinium* clade-specific primers (A to F) [83,84,85] and universal primers for Symbiodiniaceae [69,86]. Additionally, the DNA extracts were also amplified using primers designed by Palumbi et al. [87] to obtain partial plastid 23S rRNA gene sequences of *Ostreobium* sp. The amplification of ITS1-5.8S-ITS2 rRNA was performed with universal primer zITSf [86] and primer zITSr [88]. Amplification of symbiodiniaceae-specific ITS2 region was performed with primer ITSintfor2 [89] and primer ITSreverse [90], or ITS2clamp [90]. PCR of genus-specific ITS2 region was performed with seven primer pairs by [84], and genus-specific ITS1-5.8S-ITS2 rRNA for *Symbiodinium* (A) was carried out with primers designed by [83]. Amplification of 28S rRNA was performed with symbiodiniaceae-specific primers for the D1-D2 domain [69], with specific for *Breviolum*, *Cladocopium*, *Durusdinium* (B, C and D clades) designed by Correa et al. [83], with six Symbiodiniaceae genus-specific primer pairs (A to F) [85]. The primer pairs used in the reported analysis and the PCR conditions are listed in Table S4.

Amplified PCR products were electrophoresed on a 1.5–2% agarose gel and then visualized under UV illumination. The non-specific amplicons of ITS-rDNA and partial 28S rRNA were excised from the gel and then purified and cloned using a pCR4-TOPO TA cloning kit (Invitrogen, Carlsbad, CA, USA). PCR products were purified using ExoSAP-IT (Applied Biosystems, Thermo Fisher Scientific Corp., Waltham, MA, USA). Sequencing was performed using a Big Dye Terminator v3.1 Cycle Sequencing Kit (Perkin-Elmer, Foster City, CA, USA) according to the manufacturer’s protocol. After the sequencing reaction, primers and unreacted dye were removed using Centri-Sep™ columns on Sephadex G-50 (Princeton Separations, Inc. Adelphia, NJ, USA). Sequencing products were analyzed on an ABI PRISM 3500 Genetic Analyzer (Applied Biosystems, Foster City, CA, USA). Sequence chromatograms were screened for quality, edited, and assembled in the Geneious Prime software (2022.0.1, Biomatters Limited, Auckland, New Zealand). ITS2 type profiling was carried out by comparing the obtained sequences with the file containing 2564 sequences, used to define ‘defining intragenomic variants’ (DIVs) [43], and the file containing 8409 sequences from published samples, used through the SymPortal quality control pipeline and Minimum Entropy Decomposition from the SymPortal database [43]. In addition, the similarity of the obtained sequences to those of the available sequences in GenBank was determined by a BLASTn search [91]. The sequence data were deposited in GenBank under the following accession numbers: ON077166-ON077187, ON114155-ON114176, and ON142063-ON142067. The alignment was carried out using the MAFFT [92] with default settings implemented in Geneious Prime and checked by eye to exclude ambiguous regions that were difficult to align. Sequences of coral-associated organisms were identified only by their similarity with sequences from GenBank and the SymPortal database; no further phylogenetic or networks analyses were performed.

### 5.3. Lipid Analysis

Chloroform and methanol of analytical grade were used for lipid extraction. Hexane, 2-propanol, formic acid, and triethylamine of LC-MS grade (Sigma-Aldrich) were used for high-performance liquid chromatography (HPLC). The phosphatidylcholine (PC) internal standard 12:0/12:0 (850335), PG 18:0/18:1 (840503) and MGDG mixture (840523), DGDG mixture (840524) and SQDG mixture (840525) were purchased from Avanti Polar Lipids (Alabaster, AL, USA).

Total lipid extracts were obtained according to Folch et al. [93] with some modifications. A sample (~5 g) of fresh coral colonies was homogenized on a disperser (IKA T25 digital Ultra-Turrax, Staufen im Breisgau, Germany) in 10 mL of a chloroform/methanol mixture (1:2 *v*/*v*) and filtered. The residues were extracted twice with 10 mL of a chloroform/methanol mixture (2:1 *v*/*v*). The extracts were combined, mixed with chloroform (10 mL) and water (15 mL), and then left overnight for phase separation at 4 °C. Then the near-bottom layer was separated and evaporated; total lipids were dissolved in a small volume of chloroform under argon and stored at −40 °C. 

Contents and structures of GL molecular species were analyzed on a HPLC system coupled with a high-resolution tandem mass spectrometer [94]. Total lipids were separated on a Shim-Pack diol column (4.6 mm × 50 mm, particle size 5 μm) (Shimadzu, Kyoto, Japan) using a Nexera-e chromatography system (Shimadzu, Kyoto, Japan). Solvent system A (2-propanol/hexane/H_2_O/HCOOH/(28%)NH_4_OH/Et_3_N, 28/72/1.5/0.1/0.05/0.02, *v*/*v*) and solvent system B (2-propanol/H_2_O/HCOOH/(28%)NH_4_OH/Et_3_N, 100/1.5/0.1/0.05/0.02, *v*/*v*) were used as eluents. The percentage of system B in the total solvent flow was programmed as follows: 0 to 20% (7 min), 20 to 100% (5 min), 100% (5 min), 100 to 0% (0.1 min), and 0% (10 min). The elution rate was 0.2 mL/min. Lipids were detected on a high-resolution tandem mass spectrometer LCMS-IT-TOF (Shimadzu, Japan). Analysis was performed in the electrospray ionization (ESI) mode with simultaneous registration of signals of positive and negative ions. Scanning was performed in an m/z range of 100–1200. Source voltage was −3.5 kV in the case of negative ion formed and 4.5 kV in the case of positive ions formed. The temperature of the ion source was 250 °C; the dry gas (N_2_) pressure, 200 kPa; and the flow rate of nebulizing gas (N_2_), 1.5 L/ min. Argon (0.003 Pa) was used in the collision chamber of the mass spectrometer. Percentages of certain molecular species of SQDG, MGDG, and PG were calculated on the basis of the peak area of negative ions [M–H]^−^, except for DGDG, which were determined using the peak area of positive ions [M+Na]^+^ or [M+H]^+^. Molecular species were identified as described earlier [94]. For quantitative analysis, the internal PC standard 12:0/12:0 was added to aliquots of total lipid extracts. The difference in the PC, GL, and PG ionizations was taken into account using a correction coefficient. The coefficients were calculated as the ratio of the sum of the areas of individual molecular species in the standard GL mixtures (MGDG, DGDG and SQDG, separately) or the areas of individual molecular species of PG 18:0/18:1 to the area of the internal standard (PC 12:0/12:0). The peak areas of molecular species of analyzed coral lipids were calculated using the obtained coefficients. The MS/MS spectra of individual GL molecular species are represented in Figures S3 and S4.

### 5.4. Statistical Analysis

The raw data were used after being tested for normality of distribution (Shapiro–Wilk’s test). Significant differences between levels within the factors were determined by the analysis of variance (ANOVA) with the post hoc Tukey’s HSD test. A probability level of *p* < 0.05 was considered statistically significant. Values are presented as mean ± standard deviation. The preliminary data was arcsine-transformed prior to the principal component analysis (PCA) and building heat maps with cluster (tree clustering, wards method, and Euclidean distances) analyses [95]. Heat maps were composed, and all of the statistical analyses were performed using the R statistical software (“rstatix” package for ANOVA and the post hoc Tukey’s HSD test, “pheatmap” package for heat maps, “ggbiplot” package for PCA, and “cluster” and “factoextra” packages for clustering algorithms and visualization).

## Figures and Tables

**Table 1:** Results of a symbiont genetic screening in the coral colonies of *Acropora* sp. (A1–A5), *Sinularia flexibilis* (S1–S5), and *Millepora platyphylla* (M1–M5). “A1a”—*Symbiodinium*; “B1”—*Breviolum*; “C3”, “C3sg”, “C3u”, “C50bn”, “C66”, “C71/C71a”—*Cladocopium*; “D1”—*Durusdinium*.

Colony	Symbiodiniaceae Type Based on ITS Profile									*Ostreobium* sp. (23S rRNA)	Nucleotide Sequences per Coral Colony
	A1a	B1	C3	C3sg	C3u	C50bn	C66	C71/C71a	D1		
A1	+	+			+						3
A2			+	+	+		+				4
A3		+	+		+			+			4
A4	+	+	+		+	+		+		+	7
A5		+	+		+	+		+			5
M1								+	+		2
M2								+	+	+	3
M3				+				+	+	+	4
M4								+		+	2
M5			+	+				+	+	+	5
S1		+	+					+			3
S2			+								1
S3			+								1
S4			+	+				+			3
S5			+					+			2

**Table 2:** Thylakoid lipid molecular species (% of sum in lipid class) of the corals *Acropora* sp. (A1–A5), *Sinularia flexibilis* (S1–S5) and *Millepora platyphylla* (M1–M5). Abbreviations: mono- and digalactosyldiacylglycerol (MGDG and DGDG), and number of double bonds in glycolipid molecules (d.b.).

		MGDG						DGDG					
Coral Colonies		C_30–34_	C_36–38_	C_40–42_	With 0–5 d.b.	With 6–8 d.b.	With 9–11 d.b.	C_30–34_	C_36–38_	C_40–42_	With 0–5 d.b.	With 6–8 d.b.	With 9–11 d.b.
A1	Content, % of sum in lipid class	3.26	91.57	4.72	3.26	50.52	45.77	0.00	91.69	5.66	0.00	40.83	56.52
A2		5.37	84.75	4.99	5.37	45.38	44.36	0.00	87.32	8.29	0.00	35.75	59.85
A3		7.37	87.56	5.40	7.37	49.07	43.88	0.00	91.92	8.08	0.00	46.05	53.95
A4		4.67	90.68	2.93	4.67	49.24	44.37	0.00	88.91	10.74	0.00	51.60	48.05
A5		5.04	90.87	4.09	5.04	50.83	44.12	0.00	89.01	11.36	0.00	51.50	48.87
S1		63.04	36.96	0.00	42.39	41.01	16.60	43.51	55.43	2.41	29.97	45.45	25.93
S2		57.98	42.02	0.00	37.95	43.95	18.09	32.42	66.59	1.28	14.21	52.85	33.22
S3		55.34	44.66	0.00	31.13	52.78	16.09	36.04	63.01	0.95	19.58	53.69	26.73
S4		50.97	48.36	0.00	29.36	51.72	18.26	46.85	52.61	0.17	16.94	44.28	38.42
S5		53.59	46.02	0.00	33.33	48.93	17.35	43.74	55.95	0.12	16.98	50.39	32.43
M1		17.05	80.87	0.00	16.29	17.69	63.94	10.29	50.62	34.99	11.26	3.77	82.34
M2		1.53	98.29	0.00	0.32	9.32	90.18	2.61	54.73	36.47	2.61	9.34	82.82
M3		51.57	48.40	0.00	23.76	42.45	33.76	52.24	17.78	27.45	58.74	1.39	37.34
M4		40.16	60.70	0.00	32.40	24.56	43.90	27.50	49.36	23.31	31.69	5.27	63.21
M5		45.45	53.71	0.00	23.19	40.11	35.86	51.59	20.73	27.08	56.90	0.73	42.18

## Data Availability

Not applicable.

## Supplementary Materials

The following supporting information can be downloaded at https://www.mdpi.com/article/10.3390/md20080485/s1. Table S1: Profiles of the thylakoid lipid molecular species (% of total in lipid class) and content in lipid extract of the corals *Acropora* sp., *Sinularia flexibilis*, and *Millepora platyphylla*. Abbreviations are as follows: sulfoquinovosyldiacylglycerol (SQDG), mono- and digalactosyldiacylglycerol (MGDG and DGDG), and phosphatidylglycerol (PG); Figure S1: PCA analysis (additional data) of coral glycolipid molecular species grouped on the basis of double bonds: MGDG and DGDG with 0–5 double bonds, MGDG and DGDG with 6–8 double bonds, and MGDG and DGDG with 9–11 double bonds. Eigenvalues scree plot; Table S2: PCA analysis (additional data) of coral glycolipid molecular species grouped on the basis of double bonds: MGDG and DGDG with 0–5 double bonds, MGDG and DGDG with 6–8 double bonds, and MGDG and DGDG with 9–11 double bonds; Figure S2: Cluster analysis (Wards method and Euclidean distances) of coral glycolipid molecular species grouped on the basis of double bonds: MGDG and DGDG with 0–5 double bonds, MGDG and DGDG with 6–8 double bonds, and MGDG and DGDG with 9–11 double bonds. *Acropora* sp. colonies A1–A5; *Sinularia flexibilis* colonies S1–S5, and *Millepora platyphylla* colonies M1-M5; Table S3: Colonies of three tropical coral species and conditions of sample collection; Table S4: Primers and amplification conditions used in this study; Figure S3 and S4: Electrospray ionization mass spectra of 16:0/16:3,16:1/16:2, and 18:4/18:4, respectively.
