# Supplementary material for: Coral Holobionts Possess Distinct Lipid Profiles That May Be Shaped by Symbiodiniaceae Taxonomy
Source: Mar Drugs. 2022 Jul 28;20(8):485. doi: 10.3390/md20080485 (PMC9410212; doi:10.3390/md20080485)
Supplement: Supplementary file 1 [file marinedrugs-20-00485-s001.zip › marinedrugs-1743648-supplementary.pdf]

Table S1

Profiles of the thylakoid lipid molecular species (% of sum in lipid class) and content in lipid extract of the corals *Acropora sp., Sinularia flexibilis* and *Millepora phlatyphylla*. Abbreviations: sulfoquinovosyldiacylglycerol (SQDG), mono- and digalactosyldiacylglycerol (MGDG and DGDG), and phosphatidylglycerol (PG).

	Acropora sp.					*Sinularia flexibilis*					*Millepora phlatyphylla*				
**Molecular species**	**A1**	**A2**	**A3**	**A4**	**A5**	**S1**	**S2**	**S3**	**S4**	**S5**	**M1**	**M2**	**M3**	**M4**	**M5**
**PG**	**Content, % of sum in lipid class**														
32:0//	0.00	0.00	0.00	0.00	0.00	0.00	0.00	0.00	0.00	0.00	2.21	4.93	3.20	5.63	3.93
32:1//	0.00	0.00	0.00	0.00	0.00	0.00	0.00	0.00	0.00	0.00	1.44	13.40	4.21	8.78	9.24
16:0/18:1	0.00	0.00	0.00	0.00	0.00	0.00	0.00	0.00	0.00	0.00	17.01	17.56	11.84	19.78	9.58
16:0/18:2	0.00	0.00	0.00	0.00	0.00	0.00	0.00	0.00	0.00	0.00	21.30	11.96	18.64	31.82	15.08
16:1/18:2	0.00	0.00	0.00	0.00	0.00	0.00	0.00	0.00	0.00	0.00	4.40	0.16	51.70	11.22	40.73
19:2/16:1	30.66	42.10	34.70	37.73	32.01	0.00	0.00	0.00	0.00	0.00	33.04	35.98	9.06	12.43	11.41
16:0/20:1	14.41	14.42	15.70	15.44	24.74	0.00	0.00	0.00	0.00	0.00	0.00	0.00	0.00	0.00	0.00
36:2//	3.42	5.93	3.15	1.86	2.77	0.00	0.00	0.00	0.00	0.00	3.63	3.78	1.31	2.33	1.26
16:2/20:2	51.51	37.56	46.44	44.97	40.49	100.00	100.00	100.00	100.00	100.00	16.96	12.23	0.04	8.01	8.77
**SQDG**	**Content, % of sum in lipid class**														
14:0/14:0	2.72	1.69	2.50	3.57	8.69	1.68	3.06	1.67	1.21	1.30	2.10	1.03	0.14	0.91	0.66
14:2/16:0	0.00	0.00	0.00	0.00	0.00	3.53	5.68	4.46	4.49	3.58	0.00	0.00	0.00	0.00	0.00
16:0/14:1	25.26	27.90	39.73	43.47	42.97	14.80	25.20	20.29	17.92	17.12	37.11	28.78	2.66	9.95	6.07
14:0/16:0	59.71	51.83	55.43	45.92	42.00	32.79	38.38	38.33	38.91	42.88	31.79	34.76	5.25	18.25	11.93
32:4//	0.00	0.00	0.00	0.00	0.00	0.50	0.48	0.41	0.74	0.41	0.00	0.00	0.00	0.00	0.00
16:0/16:3	0.00	0.00	0.00	0.00	0.00	2.67	5.42	3.56	2.73	2.42	0.00	0.00	0.00	0.00	0.00
16:0/16:2	0.00	0.00	0.00	0.00	0.00	7.43	7.02	8.15	9.62	8.47	0.00	0.00	0.00	0.00	0.00
16:0/16:1	1.16	0.62	0.17	0.00	0.14	0.00	0.00	0.00	0.00	0.00	2.46	0.54	1.44	2.64	2.31
16:0/16:0	10.97	17.96	2.31	4.99	6.20	31.86	14.76	23.13	24.37	24.03	16.36	23.72	37.52	43.35	32.50
16:0/17:0	0.00	0.00	0.00	0.00	0.00	0.00	0.00	0.00	0.00	0.00	0.00	0.00	0.63	0.12	0.31
16:0/18:3	0.00	0.00	0.00	0.00	0.00	0.00	0.00	0.00	0.00	0.00	1.19	0.00	0.84	0.77	1.29
16:0/18:2	0.00	0.00	0.00	0.00	0.00	0.00	0.00	0.00	0.00	0.00	3.54	2.33	4.22	6.10	5.15
16:0/18:1	0.00	0.00	0.00	0.00	0.00	0.00	0.00	0.00	0.00	0.00	4.10	5.83	44.58	16.98	38.34

16:0/18:0	0.00	0.00	0.00	0.00	0.00	0.00	0.00	0.00	0.00	0.00	1.36	1.27	2.21	0.92	1.45
SQDG with PUFAs	0.00	0.00	0.00	0.00	0.00	14.13	18.60	16.58	17.58	14.88	1.19	0.00	0.84	0.77	1.29
SQDG with C_{17-18} FAs	0.00	0.00	0.00	0.00	0.00	0.00	0.00	0.00	0.00	0.00	10.18	9.44	52.48	24.90	46.53
SQDG with MUFAs	26.42	28.53	39.90	43.47	43.12	14.80	25.20	20.29	17.92	17.12	43.67	35.15	48.68	29.58	46.72
SQDG with SFAs	73.39	71.47	60.24	54.48	56.88	66.32	56.20	63.13	64.49	68.21	51.61	60.77	45.74	63.55	46.84
SQDG with C_{14-16} FAs	99.81	100.00	100.14	97.95	100.00	95.25	100.00	100.00	100.00	100.22	89.82	88.82	47.01	75.10	53.47
**MGDG**	**Content, % of sum in lipid class**														
30:0//	0.36	0.07	0.29	0.20	0.28	0.00	0.00	0.00	0.00	0.00	0.00	0.00	0.00	0.00	0.00
32:5//	0.00	0.00	0.00	0.00	0.00	3.37	2.80	3.68	2.72	3.40	0.00	0.00	0.00	0.00	0.00
32:4//	0.00	0.00	1.76	0.00	0.00	9.15	7.72	8.21	8.34	9.41	0.00	0.00	0.00	0.00	0.00
16:0/16:3	0.00	0.00	0.21	0.00	0.00	12.59	11.80	9.13	9.85	10.60	0.00	0.00	0.00	0.00	0.00
16:0/16:2	0.00	0.00	0.00	0.00	0.00	17.29	15.64	10.11	8.46	9.93	0.00	0.00	0.00	0.00	0.00
16:0/16:1	0.00	0.00	0.74	0.00	0.00	0.00	0.00	0.00	0.00	0.00	0.00	0.00	0.00	0.00	0.00
16:3/18:5	0.00	0.00	0.00	0.00	0.00	3.06	2.99	3.36	2.22	1.38	0.00	0.00	0.00	0.00	0.00
16:3/18:4	0.00	0.00	0.00	0.00	0.00	10.93	10.74	13.80	11.04	10.71	0.00	0.00	0.00	0.00	0.00
16:2/18:4	0.00	0.00	0.00	0.00	0.00	6.66	6.30	7.05	8.36	8.17	0.77	1.21	27.81	7.76	22.26
16:2/18:3	0.64	0.77	1.61	0.63	0.86	0.00	0.00	0.00	0.00	0.00	0.89	0.00	5.92	4.79	9.23
16:2/18:2	1.15	2.82	1.57	2.35	2.50	0.00	0.00	0.00	0.00	0.00	12.27	0.00	14.54	24.04	12.55
16:0/18:3	0.69	0.84	0.97	0.93	0.97	0.00	0.00	0.00	0.00	0.00	0.44	0.00	1.73	0.49	0.32
16:0/18:2	0.42	0.87	0.22	0.56	0.43	0.00	0.00	0.00	0.00	0.00	2.69	0.32	1.56	2.84	1.09
34:1//	0.00	0.00	0.00	0.00	0.00	0.00	0.00	0.00	0.00	0.00	0.00	0.00	0.00	0.24	0.00
18:5/18:5	0.00	0.00	0.00	0.00	0.00	0.00	0.00	0.00	0.00	0.00	6.79	2.33	1.31	5.79	3.54
18:4/18:5; 16:4/20:5	21.62	21.73	20.67	21.63	20.14	7.29	8.00	9.39	7.95	6.90	57.15	87.85	32.45	38.11	32.32
18:4/18:4	21.48	18.82	20.82	21.97	19.97	20.37	23.93	28.57	30.11	28.67	11.87	7.69	11.48	9.35	12.69
18:3/18:4	13.07	13.36	13.83	13.27	12.92	0.00	0.00	0.00	0.00	0.00	0.00	0.00	1.04	0.00	2.28
18:3/18:3	8.33	6.02	7.01	6.64	8.73	0.00	0.00	0.00	0.00	0.00	5.06	0.42	2.12	7.45	2.89
18:5/20:5	7.58	7.37	7.32	8.51	9.06	0.00	0.00	0.00	0.00	0.00	0.00	0.00	0.00	0.00	0.00
16:3/22:6; 18:4/20:5	11.85	10.26	10.50	11.30	10.83	9.30	10.09	6.70	10.31	10.45	0.00	0.00	0.00	0.00	0.00
18:3/20:5	7.64	7.18	7.41	7.37	9.22	0.00	0.00	0.00	0.00	0.00	0.00	0.00	0.00	0.00	0.00
18:5//22:6	1.08	1.88	1.32	0.93	1.31	0.00	0.00	0.00	0.00	0.00	0.00	0.00	0.00	0.00	0.00

18:4/22:6	2.02	2.05	2.35	1.01	1.21	0.00	0.00	0.00	0.00	0.00	0.00	0.00	0.00	0.00	0.00
18:3/22:6	0.92	0.71	1.23	0.75	1.19	0.00	0.00	0.00	0.00	0.00	0.00	0.00	0.00	0.00	0.00
20:5/22:6	0.70	0.35	0.49	0.25	0.38	0.00	0.00	0.00	0.00	0.00	0.00	0.00	0.00	0.00	0.00
C_{30-34} MGDG	3.26	5.37	7.37	4.67	5.04	63.04	57.98	55.34	50.97	53.59	17.05	1.53	51.57	40.16	45.45
C_{36-38} MGDG	91.57	84.75	87.56	90.68	90.87	36.96	42.02	44.66	48.36	46.02	80.87	98.29	48.40	60.70	53.71
C_{40-42} MGDG	4.72	4.99	5.40	2.93	4.09	0.00	0.00	0.00	0.00	0.00	0.00	0.00	0.00	0.00	0.00
MGDG 0-5 d.b.	3.26	5.37	7.37	4.67	5.04	42.39	37.95	31.13	29.36	33.33	16.29	0.32	23.76	32.40	23.19
MGDG 6-8 d.b.	50.52	45.38	49.07	49.24	50.83	41.01	43.95	52.78	51.72	48.93	17.69	9.32	42.45	24.56	40.11
MGDG 9-11 d.b.	45.77	44.36	43.88	44.37	44.12	16.60	18.09	16.09	18.26	17.35	63.94	90.18	33.76	43.90	35.86
**DGDG**							**Content, % of sum in lipid class**								
16:4/16:0; 16:2/16:2	0.00	0.00	0.00	0.00	0.00	6.93	5.17	7.74	6.73	6.21	0.00	0.00	0.00	0.00	0.00
16:0/16:3; 16:1/16:2	0.00	0.00	0.00	0.00	0.00	10.03	4.83	4.91	4.57	2.49	0.00	0.00	0.00	0.00	0.00
16:0/16:2	0.00	0.00	0.00	0.00	0.00	13.01	4.22	6.92	5.64	8.28	0.00	0.00	0.00	0.00	0.00
18:4/16:4	0.00	0.00	0.00	0.00	0.00	3.47	3.24	4.28	7.13	6.43	0.00	0.00	0.00	0.00	0.00
16:3/18:4	0.00	0.00	0.00	0.00	0.00	6.38	11.10	6.93	20.72	16.88	0.00	0.00	0.00	0.00	0.00
16:2/18:4	0.00	0.00	0.00	0.00	0.00	3.68	3.86	5.25	2.07	3.45	0.00	0.00	0.00	0.00	0.00
18:2/16:2	0.00	0.00	0.00	0.00	0.00	0.00	0.00	0.00	0.00	0.00	2.08	0.90	11.64	3.45	7.06
18:2/16:1	0.00	0.00	0.00	0.00	0.00	0.00	0.00	0.00	0.00	0.00	0.00	0.00	3.59	1.60	3.18
18:2/16:0	0.00	0.00	0.00	0.00	0.00	0.00	0.00	0.00	0.00	0.00	7.63	1.71	28.58	18.78	34.77
18:1/16:0	0.00	0.00	0.00	0.00	0.00	0.00	0.00	0.00	0.00	0.00	0.59	0.00	8.43	3.68	6.58
18:4/18:5	15.96	9.57	13.72	0.77	7.86	3.24	6.22	2.84	10.37	8.34	22.52	23.55	4.48	18.66	7.12
18:4/18:4	22.12	15.61	20.05	6.07	8.91	31.91	34.64	37.23	14.36	23.63	3.77	7.76	1.39	5.27	0.73
18:3/18:4	9.61	5.57	6.15	13.97	9.65	0.00	0.00	0.00	0.00	0.00	0.00	0.00	0.00	0.00	0.00
18:3/18:3	0.90	3.24	4.71	5.86	7.77	0.00	0.00	0.00	0.00	0.00	0.00	1.58	0.00	0.00	0.00
36:5//	0.00	0.00	0.00	0.00	0.00	0.00	0.00	0.00	0.00	0.00	0.44	0.00	0.00	0.24	0.37
18:2/18:2	0.00	0.00	0.00	0.00	0.00	0.00	0.00	0.00	0.00	0.00	0.48	0.00	5.27	3.60	4.32
36:3//	0.00	0.00	0.00	0.00	0.00	0.00	0.00	0.00	0.00	0.00	0.04	0.00	1.23	0.35	0.62
18:5/20:5	13.75	15.14	14.45	7.91	10.27	2.86	4.55	3.08	0.20	1.42	8.56	9.31	3.69	14.09	2.64
18:4/20:5	21.15	26.86	17.70	28.62	19.38	17.42	21.18	19.86	27.68	22.56	14.81	12.53	1.71	7.15	4.93
18:3/20:5	8.20	11.34	15.14	25.70	25.17	0.00	0.00	0.00	0.00	0.00	0.00	0.00	0.00	0.00	0.00

20:5/19:5	0.00	0.00	0.00	0.00	0.00	0.00	0.00	0.00	0.00	0.00	1.46	0.96	0.00	0.00	0.41
18:4/22:6	2.71	4.95	2.62	3.66	2.86	2.41	1.28	0.95	0.17	0.12	26.45	27.87	21.12	16.46	19.05
18:3/22:6	1.59	0.59	2.72	3.90	4.83	0.00	0.00	0.00	0.00	0.00	0.35	0.00	0.00	0.00	0.00
20:5/22:6	1.35	2.75	2.74	3.18	3.67	0.00	0.00	0.00	0.00	0.00	8.19	8.61	6.33	6.85	8.03
C_{30-34} DGDG	0.00	0.00	0.00	0.00	0.00	43.51	32.42	36.04	46.85	43.74	10.29	2.61	52.24	27.50	51.59
C_{36-38} DGDG	91.69	87.32	91.92	88.91	89.01	55.43	66.59	63.01	52.61	55.95	50.62	54.73	17.78	49.36	20.73
C_{40-42} DGDG	5.66	8.29	8.08	10.74	11.36	2.41	1.28	0.95	0.17	0.12	34.99	36.47	27.45	23.31	27.08
DGDG 0-5 d.b.	0.00	0.00	0.00	0.00	0.00	29.97	14.21	19.58	16.94	16.98	11.26	2.61	58.74	31.69	56.90
DGDG 6-8 d.b.	40.83	35.75	46.05	51.60	51.50	45.45	52.85	53.69	44.28	50.39	3.77	9.34	1.39	5.27	0.73
DGDG 9-11 d.b.	56.52	59.85	53.95	48.05	48.87	25.93	33.22	26.73	38.42	32.43	82.34	82.82	37.34	63.21	42.18
**Lipid class**	**Content, % of lipid extract**														
**PG**	0.1469	0.0440	0.0867	0.0219	0.0594	0.0039	0.0062	0.0060	0.0053	0.0031	0.0253	0.0123	0.1440	0.0690	0.0742
**SQDG**	6.8812	2.0479	4.9016	0.8488	1.5265	1.6173	0.8970	0.4970	1.0035	0.8697	1.6642	0.8604	5.8907	6.1965	13.8563
**MGDG**	4.8600	2.2676	5.3240	1.9574	3.6616	3.0129	3.0175	1.8573	3.8879	1.9384	2.2169	1.2342	2.2867	2.8618	2.8500
**DGDG**	4.8848	3.9898	6.1799	4.2784	6.4828	2.2608	2.2917	1.7600	0.9912	0.8608	3.2406	3.4490	3.3638	3.0186	2.9500
**Lipid class**	**Ratio of lipids**														
SQDG/PG	46.85	46.51	56.55	38.70	25.71	415.95	144.86	82.32	188.52	278.71	65.79	69.80	40.92	89.76	186.68
DGDG/MGDG	1.01	1.76	1.16	2.19	1.77	0.75	0.76	0.95	0.25	0.44	1.46	2.79	1.47	1.05	1.04

PG, MGDG, DGDG, SQDG: *sn*-1(2) acyl/ *sn*-2(1) acyl

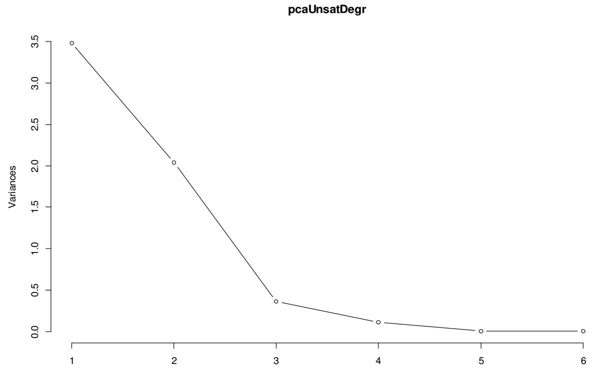

Figure S1 PCA analysis (additional data) of coral glycolipid molecular species grouping on the basis of double bounds: MGDG and DGDG with 0-5 double bounds, MGDG and DGDG with 6-8 double bounds, and MGDG and DGDG with 9-11 double bounds. Eigenvalues scree plot.

Table S2

PCA analysis (additional data) of coral glycolipid molecular species grouping on the basis of double bounds: MGDG and DGDG with 0-5 double bounds, MGDG and DGDG with 6-8 double bounds, and MGDG and DGDG with 9-11 double bounds.

			Rotation (n x k) = (6 x 6):			
	PC1	PC2	PC3	PC4	PC5	PC6
0-5 MGDG	0.4041129	-0.392450685	-0.53531929	0.3424240	-0.32437155	-0.41669814
6-8 MGDG	0.4161161	0.347296147	0.60637627	0.4007562	0.05741072	-0.41789838
9-11 MGDG	-0.5312046	0.020906151	0.07762616	-0.3328979	-0.20742735	-0.74666705
0-5 DGDG	0.2012324	-0.639215860	0.21590517	-0.2716096	0.62747239	-0.19183368
6-8 DGDG	0.2689449	0.562414009	-0.50714659	-0.2989807	0.46337010	-0.22374128
9-11 DGDG	-0.5182046	-0.004934181	-0.18947966	0.6694460	0.48992606	-0.08574148
			Importance of components:			
Standard deviation	1.86620356	1.42684198	0.60095747	0.33601517	0.06862602	0.05138728
Proportion of Variance	0.5805	0.3393	0.06019	0.01882	0.00078	0.00044
Cumulative Proportion	0.5805	0.9198	0.97996	0.99877	0.99956	1.00000

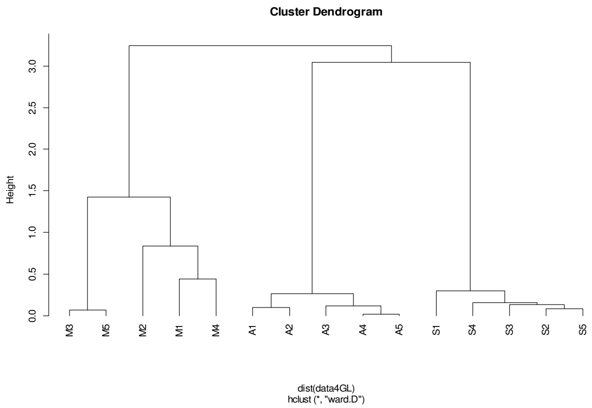

Figure S2 Cluster analysis (Wards method and Euclidean distances) of coral glycolipid molecular species grouping on the basis of double bounds: MGDG and DGDG with 0-5 double bounds, MGDG and DGDG with 6-8 double bounds, and MGDG and DGDG with 9-11 double bounds. *Acropora* sp. colonies A1-A5; *Sinularia flexibilis* colonies S1-S5 and *Millepora phlatyphylla* colonies M1-M5.

Table S3

Colonies of three tropical coral species and sample collection conditions.

Coral species	***Acropora* sp.**	***Sinularia flexibilis***	***Millepora phlatyphylla***
Image	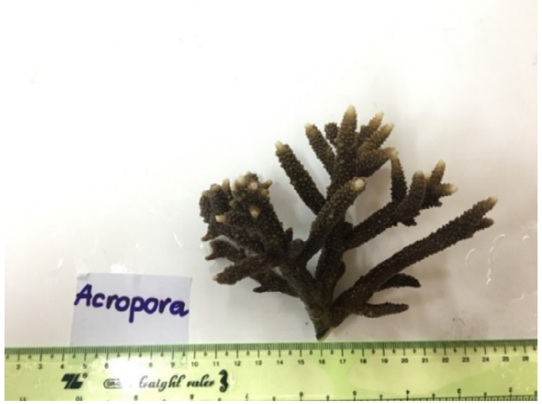	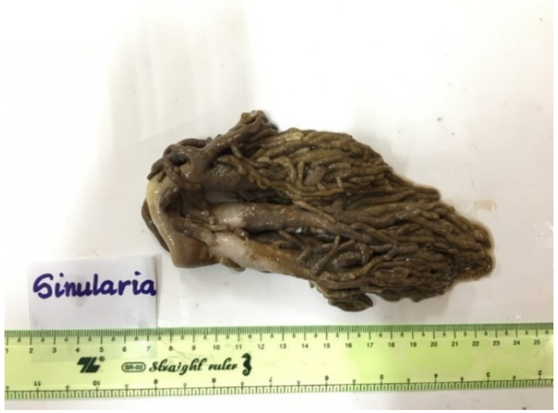	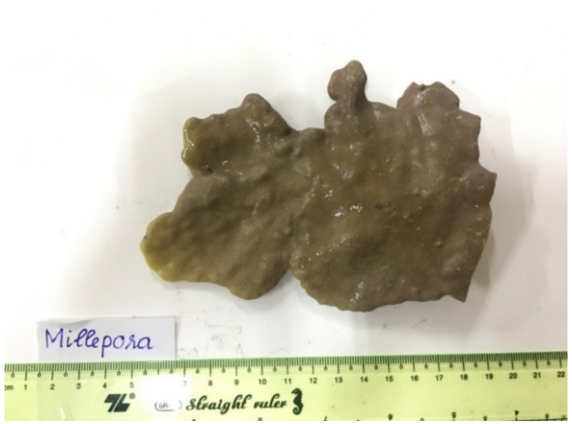
Location of sampling	Hon Tam, South China Sea; 12.174820°, 109.238961°	Hon Mot, South China Sea; 12.181341°, 109.276905°	Hon Tre, South China Sea; 12.180763°, 109.283778°
Temperature, °C	26.4	27.2	27.0
Depth, m	3.0	7.5	8.1
DO, mg/l	6.57	6.65	6.85
Salinity, %	33.57	33.44	33.49
Date, d.m.y	04.04.2021	04.04.2021	04.04.2021

The annual range condition in Nha Trang

The time of year with warmer water lasts for 2.4 months, from August 2 to October 16, with an average temperature above 28°C. The month of the year in Nha Trang with the warmest water is September, with an average temperature of 29°C. The time of year with cooler water lasts for 2.6 months, from December 17 to March 5, with an average temperature below 25°C. The month of the year in Nha Trang with the coolest water is January, with an average temperature of 25°C. The average surface water temperature in Nha Trang is *increasing* during the spring, *rising* by *3°C*, from *25°C* to *29°C*, over the course of the season. The highest average surface water temperature during the spring is *29°C* on *May 22*. The average surface water temperature in Nha Trang is gradually increasing during April, rising by 2°C, from 27°C to 28°C, over the course of the month.
https://weatherspark.com/y/120736/Average-Weather-in-Nha-Trang-Vietnam-Year-Round#Figures-WaterTemperature

The average daily incident shortwave solar energy in Nha Trang is very rapidly decreasing during the spring, falling by 2.5 kWh, from 6.4 kWh to 3.9 kWh, over the course of the season. The highest average daily incident shortwave solar energy during the spring is 6.6 kWh on March 23. The brighter period of the year lasts for 2.4 months, from February 14 to April 26, with an average daily incident shortwave energy per square meter above 6.1 kWh. The brightest month of the year in Nha Trang is March, with an average of 6.6 kWh. The darker period of the year lasts for 5.0 months, from May 21 to October 21, with an average daily incident shortwave energy per square meter below 4.4 kWh. The darkest month of the year in Nha Trang is June, with an average of 3.8 kWh. The average daily incident shortwave solar energy in Nha Trang is gradually decreasing during April, falling by 0.7 kWh, from 6.6 kWh to 5.9 kWh, over the course of the month. https://weatherspark.com/y/120736/Average-Weather-in-Nha-Trang-Vietnam-Year-Round#Figures-SolarEnergy

Table S4
Primers and amplification conditions used in this study.

Gene	Primer name	Primer sequence (5'⟶3')	Program	Reference
		Symbiodiniaceae		
28S rRNA (D1-D2)	28Szoox-D1/D2F	CCTCAGTAATGGCGAATGAACA	96°C - 3 min (96°C 60s, 55°C 60s, 72°C 120s) x5, (96°C 30s, [55→50]°C 60s, 72°C 120s)x30, 72°C 7 min	Loh et al., 2001
	28Szoox-D1/D2R	CCTTGGTCCGTGTTTCAAGA		
ITS2	ITSintfor2	GAATTGCAGAACTCCGTG	96°C - 3 min (96°C 30s, [62→52]°C 30s, 72°C 120s) x20, (92°C 30s, 52°C 30s, 72°C 120s) x20, 72°C 10 min	LaJeunesse and Trench, 2000
	ITS2clamp	CGCCCGCCGCGCCCCGCGCCCGTCCCGCGG GATCCATATGCTTAAGTTCAGCGGGT		
	or			
	ITS-Reverse	GGGATCCATATGCTTAAGTTCAGC GGGT		Coleman et al., 1994
ITS region	zITSf	CCGGTGAATTATTCGGACTGACGCAGTGCT		Hunter et al., 1997
	zITSr	TCCTCCGCTTATTGATATGC		White et al., 1990
ITS region clade A-specific	A (forward)	CCTCTTGGACCTTCCACAAC	96°C - 5 min (96°C 30s, 60°C 30s, 72°C 60s) x38, 72°C 5 min	Correa et al., 2009
	A (reverse)	GCATGCAGCAACACTGCTC		
28S rRNA (D2) clade B-specific	B (forward)	GTCTTTGTGAGCCTTGAGC		
	B (reverse)	GCACACTAACAAGTGTACCATG		
28S rRNA (D2) clade C-specific	C (forward)	CTTGAAATCGCTGAAAGGGA		
	C (reverse)	CTATTCACGCTTAAGCACACA		
28S rRNA (D2) clade D-specific	D (forward)	GCCGTGTACGGTGCTCGCTCTCAA		
	D (reverse)	GGCCACTCGCAAATGGACAGC		
ITS2 A-specific	S.S. ITS2 F	TTCTGCTGCTCTTGTTATCAGG	96°C - 3 min (96°C 30s, [62→52]°C 30s, 72°C 120s) x20, (92°C 30s, 52°C 30s, 72°C 120s) x20, 72°C 10 min	Saad et al., 2020
	S.S. ITS2 R	ACACACATGAGCTTTTGTTTCG		
ITS2 B-specific	S.B. ITS2 F	GCAAGCAGCATGTATGTC		
	S.B. ITS2 R	CTTGGAACAACAGTACGCTC		
ITS2 C-specific	S.C. ITS2 F	TGCGTTCTTATGAGCTATTGCC		
	S.C. ITS2 R	CAGCGTCACTCAAGTAAAACCA		
ITS2 D-specific	S.D. ITS2 F	TTTGCTTCAGTGCTTATTTTACCT		
	S.D. ITS2 R	ACGGCGCAGAAGGACAC		
ITS2 E-specific	S.E. ITS2 F	GAGGTAAGCTGGACTGATTTG		
	S.E. ITS2 R	TTAGTTCCTTTTCCTCCGCT		
ITS2 F-specific	S.F. ITS2 F	CCTGTGAGCCATTGAAACTCTAGT		
	S.F. ITS2 R	CAGCGTCACTCAAGAAATACCAT		

ITS2 G-specific	S.G. ITS2 F	CAGTGCAATGCCTCCTTGTG		
	S.G. ITS2 R	CCCACGCATATTCCGGAGA		
28S rRNA (D2) clade A-specific	SymA-28S F	GATTGTGGCCTTTAGACATACTACC	96°C - 3 min (96°C 30s, [62→55]°C 30s, 72°C 120s) x20, (92°C 30s, 55°C 30s, 72°C 120s) x20, 72°C 10 min	Yamashita et al. 2011
	SymA-28S R	CTCTGAGAGCAAGTACCGTGC		
28S rRNA (D2) clade B-specific	SymB-28S F	CACATGTCGTGCTGAGATTGC		
	SymB-28S R	CTCGCATGCTGAGAAACACTG		
28S rRNA (D2) clade C-specific	SymC-28S F	TTGCTGAGATTGCTGTAGGCT		
	SymC-28S R	TCCTCAAACAGGTGTGGC		
28S rRNA (D2) clade D-specific	SymD-28S F	AATGCTTGTGAGCCCTGGTC		
	SymD-28S R	AAGGCAATCCTCATGCGTATG		
28S rRNA (D2) clade E-specific	SymE-28S F	CGAGTTTTCACTAGCCTTGTGTG		
	SymE-28S R	AGCGTTGCAGCTGACGAG		
28S rRNA (D2) clade F-specific	SymF-28S F	ACAGATCTTGCTGAGATTGCTGTG		
	SymF-28S R	GAAGGCCGTCCTCAAACAGAC		
		Ostreobium (Bryopsidales, Ulvophyceae)		
23S rRNA	16Sar*	CGCCTGTTTATCAAAAACAT	96°C - 5 min (96°C 30s, 55°C 30s, 72°C 60s) x38, 72°C 5 min	Palumbi et al. 1996
	16Sbr*	CCGGTYTGAACTCAGATCAYGT		

* - Universal primers that were designed to amplify a partial 16S mtDNA are able to amplify another partial plastid gene, the 23S rRNA. The result from this is a cross homology of primers to other region of the template strand, and the outcome is amplification of other genes outside the region of target (https://www.benchling.com/primers/).

16:0/16:3; 16:1/16:2 DGDG molecular species with RT = 12.30 min gave positive ions $[M+Na]^+$ at m/z 909.553 corresponding to the composition $[C_{47}H_{82}O_{15}+Na]^-$ (calculated 909.555) (Fig. S3(b)). MS^2 fragmentation of these ions (Fig. S3(c)) resulted in the formation of ions at *m/z* 659.350 ($[C_{31}H_{56}O_{13}+Na]^+$), *m/z* 657.341 ($[C_{31}H_{54}O_{13}+Na]^+$) and *m/z* 653.312 ($[C_{31}H_{52}O_{13}+Na]^+$), corresponding to elimination of 16:3, 16:2 and 16:0 acyl fragment, respectively. According to the elemental composition calculated and the value of monoisotopic molecular mass, this component was identified as 16:0/16:3; 16:1/16:2 DGDG.

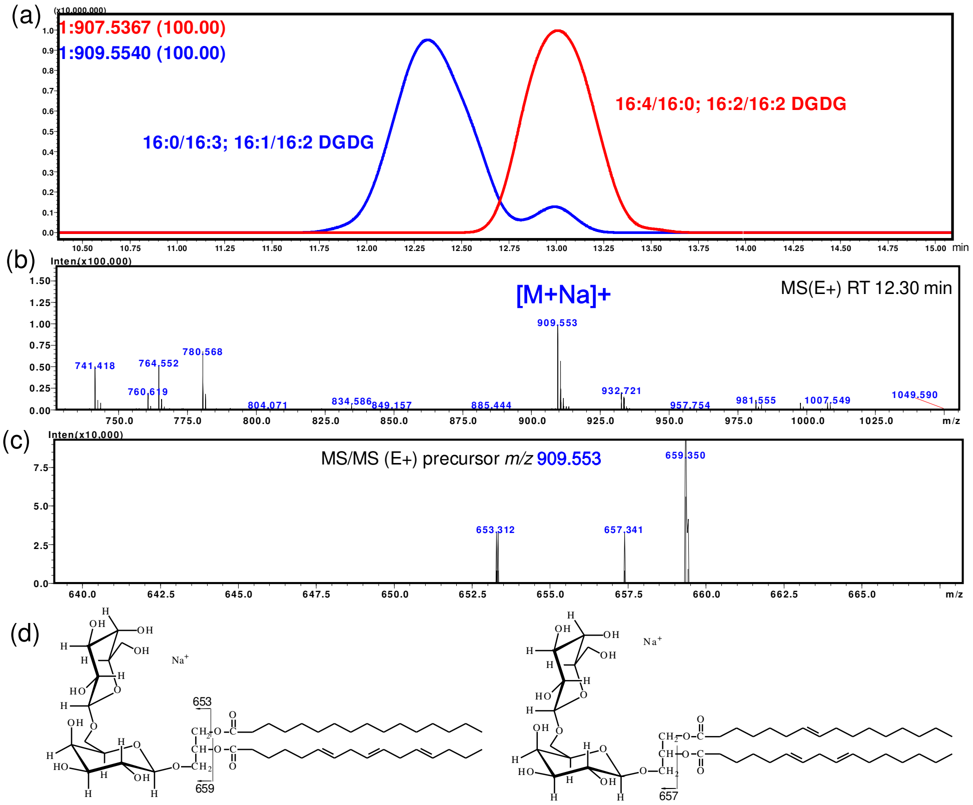

Figure S3 Electrospray ionization mass spectra of 16:0/16:3; 16:1/16:2 DGDG. The total lipids of *S. flexibilis* corals were analyzed using normal phase liquid chromatography – high resolution tandem mass spectrometry (LC–HRMS) detecting negative and positive ions. Total ion current chromatogram of two molecular species DGDG (16:0/16:3; 16:1/16:2 DGDG and 16:4/16:0; 16:2/16:2 DGDG) are presented (**a**). The mass spectra for 16:0/16:3; 16:1/16:2 DGDG eluting between 11.8-12.8 min (**b**). The MS/MS spectra of *m/z* 909.553 (**c**) are presented. The predicted structures of 16:0/16:3 DGDG and 16:1/16:2 DGDG, and the product ions are shown (**d**).

18:4/18:4 DGDG molecular species with RT = 13.53 min gave positive ions [M+Na]+ at m/z 955.534 corresponding to the composition [C51H80O15+Na]+ (calculated 955.539) (Fig. S4(b)). MS2 fragmentation of these ions (Fig. S4(c)) resulted in the formation of ions at m/z 679.322 ([C33H52O13+Na]+) and m/z 517.275 ([C33H52NaO13 – C6H11O5+Na]+), corresponding to elimination of 18:4 acyl fragment and 18:4 acyl fragment and galactose fragment, respectively. Additional ions m/z 405.141 ([C14H23O12+Na]+) and m/z 347.101 ([C12H20O10+Na]+) corresponded to galactose-containing fragments. According to the elemental composition calculated and the value of monoisotopic molecular mass, this component was identified as 18:4/18:4 DGDG.

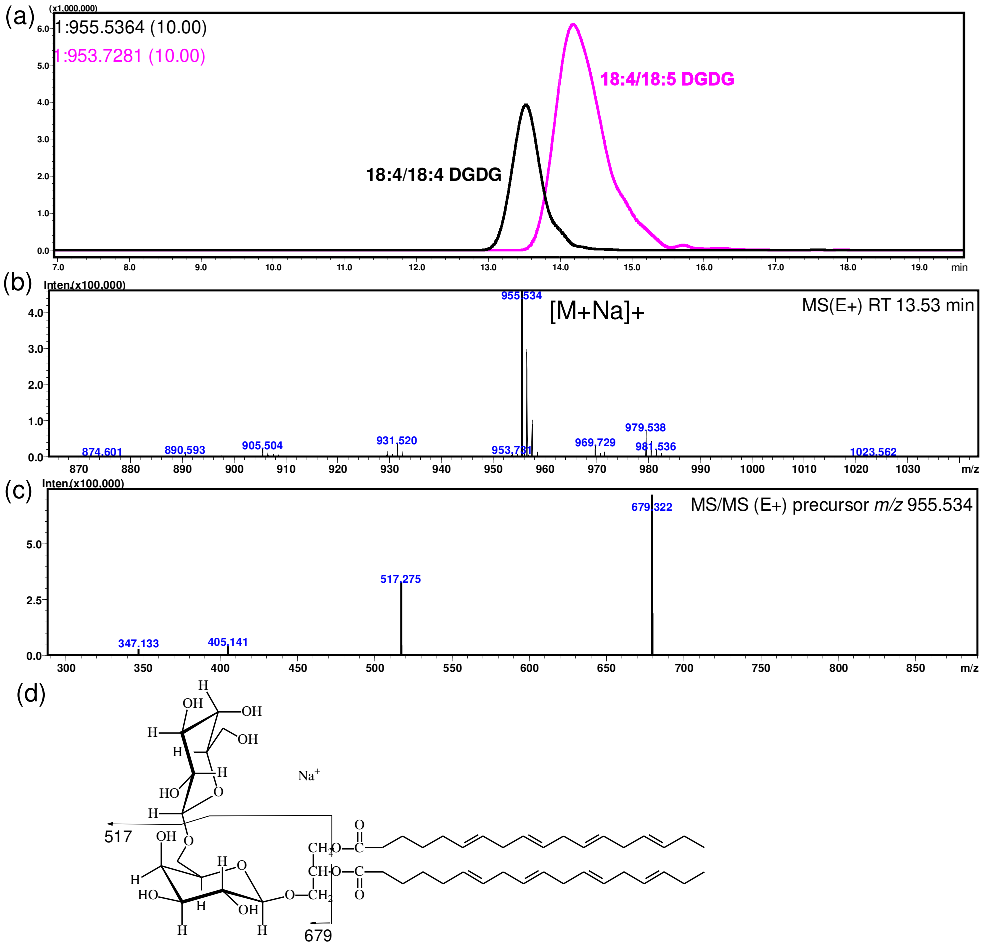

Figure S4 Electrospray ionization mass spectra of 18:4/18:4 DGDG. The total lipids of S. flexibilis corals were analyzed using normal phase liquid chromatography – high resolution tandem mass spectrometry (LC–HRMS) detecting negative and positive ions. Total ion current chromatogram of two molecular species DGDG (18:4/18:4 DGDG and 18:4/18:5 DGDG) are presented (a). The mass spectra for 18:4/18:4 DGDG eluting between 13.0-14.0 min (b). The MS/MS spectra of m/z 955.534 (c) are presented. The predicted structures of 18:4/18:4 DGDG, and the product ions are shown (d).
